# Respecting Older Adults: Lessons from the COVID-19 Pandemic

**DOI:** 10.1007/s11673-021-10164-6

**Published:** 2022-01-27

**Authors:** Cristina Voinea, Tenzin Wangmo, Constantin Vică

**Affiliations:** 1 Department of Philosophy and Social Sciences, Bucharest University of Economic Studies, Căderea Bastilliei 1, Bucharest, Romania; 2 Institute for Biomedical Ethics, University of Basel, Bernoullistrasse 28, Basel, Switzerland; 3 Department of Philosophy, University of Bucharest, Splaiul Independenței 204, Bucharest, Romania

**Keywords:** Respect, Older adults, Well-being, COVID-19, Redress

## Abstract

The COVID-19 pandemic has exacerbated many social problems and put the already vulnerable, such as racial minorities, low-income communities, and older individuals, at an even greater risk than before. In this paper we focus on older adults’ well-being during the COVID-19 pandemic and show that the risk-mitigation measures presumed to protect them, alongside the generalization of an ageist public discourse, exacerbated the pre-existing marginalization of older adults, disproportionately affecting their well-being. This paper shows that states have duties to adopt and put into practice redress measures to compensate for the negative consequences of COVID-19 public health policies on older adults’ overall well-being. These duties flow from the minimal ethical requirement of respect for persons. We show that respect is a morally basic attitude that presupposes taking the others’ interests into account, with the aim of advancing their well-being. This duty is not limited to kinship, relatives, and friends but it extends to states and the rest of the civil society. In the conclusion, we draw lessons from the COVID-19 pandemic and sketch some redress measures that could compensate for the decrease in older adults’ well-being as a result of the adoption of measures to contain the spread of the virus.

The COVID-19 pandemic was and still is a profoundly disruptive event. It has changed how we work, travel, and interact with others, and has also brought to the fore the cracks and injustices in our societies. Although it has been called “the great leveler,” because it affects all people, regardless of their social status or wealth, COVID-19 exacerbated many social problems and put the already vulnerable and marginalized, such as racial minorities, low-income communities and older individuals, at an even greater risk than before (Hay 2020).

Even though age-stratification of disease risk is not uncommon in epidemiological research (Fletcher, 2020), the current pandemic has brought to the front of public discussions the problem of ageism. Ageism is not a new problem; in fact, the World Health Organization (WHO) stated that ageism is one of the last widely socially accepted and strongly institutionalized forms of prejudice (World Health Organization 2020). It is extremely easy to find examples of ageism in the risk mitigation measures against the COVID-19 pandemic, from age-based rationing of medical resources, to seeing the pandemic as primarily “an older people” problem or excluding nursing home residents from death counts (Barrett, Michael, and Padavic 2020; Ayalon et al. 2020; Previtali, Allen, and Varlamova 2020; Fraser et al. 2020). What catalysed the ample and still ongoing debates regarding the ageism inherent in the measures for containing the spread of the virus is governments’ responses regarding age-based isolation. Older adults were and still are instructed to maintain stricter social isolation than other individuals, and for a longer period, in spite of all the risks this can entail for their overall well-being (Reynolds 2020; Fletcher, 2020; Cox 2020; Brooke and Jackson 2020).

The risk mitigation measures adopted in most states, such as physical distancing and the severe reduction of social contact disproportionately affected older adults and their well-being. The claim of this paper is that states have duties to adopt and put into practice redress measures to compensate for the negative consequences of anti-COVID-19 public health policies on older adults’ overall well-being. These duties flow from the minimal ethical requirement of respect for persons. We start by exploring how older adults’ well-being was negatively affected during the COVID-19 pandemic, mainly through the risk mitigation measures employed by most states, which subjected them to social isolation, loneliness, and their associated harms but also through the rhetoric of vulnerability used by public institutions and individuals alike. These measures, although premised on respect and value for their lives, paradoxically backfired by minimizing a risk (contracting SARS-CoV-2), while increasing another (the negative health outcomes as a result of social isolation and reinforced ageism) (Smith, Steinman, and Casey 2020). Thereafter, we delineate a substantive conception of respect. We stress that respect is a morally basic attitude that presupposes taking the others’ interests into account, with the aim of advancing their well-being. The next section claims the minimal ethical requirement is not limited to kinship, relatives, and friends, extending to states and the rest of the civil society. We show that if indeed states have duties to treat every citizen with respect, then governments are under moral obligations to put in place redress measures to compensate for the significant decrease in older adults’ well-being. In the conclusion, we draw lessons from the COVID-19 pandemic and sketch some redress measures.

## The Impact of COVID-19 Risk Mitigation Measures on Older Adults’ Well-being

Measures for pandemic control from governmental agencies conflate age with extreme vulnerability to confine older individuals at home or in nursing homes in order to mitigate risks. Such measures have polarized researchers and public opinion. Some claim because those who are sixty-five years and older compose a heterogeneous group with differences in cognitive and psychological performance, personality, social needs, cultural background, genetics and, more generally, somatic functioning (Ehni and Wahl 2020; Fingerman and Trevino 2020), selectively isolating older adults and treating them as if they were equal by imposing blanket mitigation measures is unjustified (Fraser et al. 2020; Barrett, Michael, and Padavic 2020; Ehni and Wahl 2020). The other camp maintains that from an ethical point of view, selective isolation of *all* older individuals is ethically permissible, because it is proportionate—in that it brings benefits for both the elderly persons (minimizing their mortality) and for society at large (an indiscriminate lockdown would gravely affect the economy which would affect everybody’s well-being). What’s more, opposing it would presuppose “levelling down equality” (Savulescu and Cameron 2020), which is deemed morally repulsive.

Discussing the moral permissibility of the COVID-19 mitigation measures focused exclusively on older adults is beyond the scope of this paper. However, we must acknowledge that there was and still is no perfect way to successfully “shield” older adults, while allowing the rest of the population to go about their lives normally. Believing the contrary would be premised on a naïve view of older adults’ role and place in communities—they are not somewhere at the margins but deeply interconnected with the rest of the population, as we will show below. Unfortunately, most governments assumed that physical distancing could protect older adults, which once again shows the misunderstanding of their social status and worth. The debates concerning the moral permissibility of age-stratification for risk-mitigation measures, which are controversial and still ongoing, are different from the debates regarding the overall decrease of older adults’ well-being flowing from these measures. Our preoccupation in this paper is with the latter problem. Even if states had no other choice but to impose stricter measures regarding older adults, one cannot escape the problem of the accentuated social exclusion of and prejudice against older adults that arose as unintended side-effects of risk mitigation measures, which might affect their well-being for years to come. In what follows we show what are some of the unintended consequences of the emergency public health measures aimed at “shielding” older adults.

One common worldwide response to the pandemic was to issue stay at-home orders to older adults and to recommend cutting off social contact as much as possible (D’Cruz and Banerjee 2020). But older adults, despite being retired, participate in the workforce, be it paid or unpaid, provide informal care for grandchildren and others and are an active part of civil society (Previtali et al. 2020). This policy stance gave rise to the “social connectivity paradox” (Smith, Steinman, and Casey 2020), where a set of measures devised to protect older individuals backfired by exposing them to another type of harm, different from the COVID-19 disease. More precisely, as the amount of social interaction decreases for older individuals, they are more protected from contracting SARS-CoV-2, and these are the intended consequences of the risk mitigation measures. But they nonetheless become more exposed to the fear, stress, loneliness, and anxiety stemming from home-confinement measures and social isolation, which in the end affect their resilience, by impacting both physical and mental health, and these are the unintended consequences of the same measures (Kowsalya 2020; Kessler and Bowen 2020; Armitage and Nellums 2020; Grossman et al. 2021). And while this was true for everybody during the “first wave” of the pandemic, when general lockdown measures were imposed, it is especially more troubling in the case of older adults who are the subjects of social isolation and loneliness that long precedes the COVID-19 pandemic (Lamprini 2016). In fact, social isolation of older individuals has long been considered a “serious public health concern” (Armitage and Nellums 2020) due to its consequences, such as cardiovascular, autoimmune, neurocognitive, and mental health problems (Gerst-Emerson and Jayawardhana 2015). In other words, home-confinement measures and physical distancing worsened the pre-existing loneliness and isolation that older adults are subject to. Even older adults who had a socially active life are disproportionately affected by demands to avoid social contact as much as possible, inasmuch as they are not able to maintain a minimal level of social activity necessary for preventing feelings of isolation and loneliness (Brooke and Jackson 2020). Moreover, social isolation of older adults, especially in nursing homes, has also increased their vulnerability to abuse and violence (Han and Mosqueda 2020), with some preliminary studies reporting a tenfold increase in reports of elder abuse during the pandemic (D’Cruz and Banerjee 2020).

Besides the direct consequences of social isolation on older adults’ well-being, they also suffer the indirect consequences of a rhetoric reinforcing negative age-based stereotypes or, to put it simply, ageism. Ageism, which is generally experienced as a lack of respect or a series of incorrect assumptions (Chasteen, Horhota, and Crumley-Branyon 2020), irrespective of its manifestation as benevolent or hostile prejudice (Cary, Chasteen, and Remedios 2017; Chonody 2016; North and Fiske 2012), is rampant worldwide and results in the marginalization and exclusion of older individuals from social life (Burnes et al. 2019). Older people are associated with debilitation, they are deemed not worthy of attention, non-competitive or unsuitable for employment, although warm (Chang et al. 2020; Cherry and Palmore 2008; Cuddy et al. 2005). Thus, ageism can be both benevolent and hostile (Apriceno et al. 2020). Unfortunately, these stereotypes are largely consistent across cultures and they most often result in social exclusion (Inglehart et al. 2014; Stuckelberger et al. 2013; Walsh et al. 2017).

The utilitarian cost-benefit analysis that justified prioritizing the young over older adults, contributed to the normalization of ageist beliefs (D’Cruz and Banerjee 2020) and this was most visible on social media, where hashtags such as “boomer remover” and “boomer doomer” became popular during the pandemic (Meisner 2020; Soto-Perez-de-Celis 2020; Jimenez-Sotomayor, Gomez-Moreno, and Soto-Perez-de-Celis 2020). One study conducted in three countries with different responses to the COVID-19 pandemic (Australia, the United Kingdom, and the United States) identifies a common accentuation of a rhetoric of disposability and blame in mass-media that casts “older adults as a problem to be ignored or solved through segregation” (Lichtenstein 2020). It seems that in all three countries the indefinite isolation of older adults was widely accepted as a trade-off for ending generalized lockdowns and economic crises. A recent meta-analysis shows that merely being exposed to prejudices regarding old age can have the potential to negatively influence psychiatric conditions in older adults (Chang et al. 2020). What is more, as a result of pervasive ageism in public discourse, older people internalize the idea that other people are more suitable to decide on measures for their well-being, which can ultimately lead to reducing their autonomy and control over their own lives and health (Kessler and Bowen 2020; Rahman and Jahan 2020).

The risk-mitigation measures presumed to protect older individuals, alongside the generalization of an ageist public discourse exacerbate pre-existing marginalization of older persons during the pandemic leading to “an invisible human rights crisis” (D’cruz and Banerjee 2020, 1) that can result in a decrease of older adults’ well-being (Ayalon et al. 2020; Fraser et al. 2020; Miller 2020). Well-being is a “thick concept” (Williams 2006) which means it has descriptive content but at the same time it is also evaluatively loaded. This is why there are multiple theories of well-being, which differ on a range of dimensions such as the values they refer to or the meanings or understandings of well-being (Søraker et al. 2015). But one aspect that most theories and empirical studies identify as constitutive of well-being is autonomy, the capacity to live one’s life according to one’s own reasons, motives, and beliefs, unhampered by external distorting forces (Reis et al. 2000; Ryff and Keyes 1995; Wichmann 2011; Toledano-González, Labajos-Manzanares, and Romero-Ayuso 2019; Pethtel, Moist, and Baker 2018). This independence is necessary in order for people to positively evaluate their lives and to find significance and meaning in them. Individuals do not just have the kinds of supportive and positive social relationships necessary for their well-being with their next of kin but also with members of society in general (Forsman et al. 2013; Merz and Huxhold 2010; Huxhold, Miche, and Schüz 2014).

The COVID-19 risk mitigation measures backfired in the case of older adults precisely with respect to these two elements central to almost all theories on well-being i.e., autonomy and social relationships. Firstly, home confinement and self-isolation measures disproportionately affected older adults as they were already socially excluded even before the pandemic, due to deeply entrenched ageism. Moreover, the worldwide “grey digital divide,” further prevented some older adults from maintaining even a semblance of social connections that younger generations could enjoy (Lawrence and Harris 2021). Also, the flurry of public discourses framing older adults as vulnerable can be frightening and fear-inducing, increasing feelings of anxiety and fear (Flett and Heisel 2020). The exposure to and internalization of ageism increases the risk of conforming to negative stereotypes which was shown to “reduce autonomy, independence, and quality of life” (Swift et al. 2017; Storlie 2015). So, it is not just a higher mortality from SARS-CoV-2 that affects older adults’ well-being; this tendency is doubled by the decrease of social contacts and ageist public discourses. Our claim is that states’ lack of response regarding the decrease in older adults’ well-being as a result of social isolation and reified ageism during the pandemic implies a lack of respect towards them.

## Respect as Paying Attention

Old age and ageing are not and never were the flavour of the month in bioethics, even though most of us hope to grow as old as possible. Usually, ageing and the status of older adults are addressed in the frameworks of public health ethics—what to do with the growing and increasingly expensive older population—end-of-life decisions, or nursing ethics. It almost seems that older adults are more like subjects, rather than agents of ethical discourses, as if most decision regarding their lives and well-being must be taken by others and only borne out by them (Wareham 2018). Indeed, the concerns, lives, and value of older adults are usually framed within a deficit model where the “aging person becomes someone who has not quite got what it takes to be a standard bioethical agent” (Holm 2013, 68). This does not necessarily imply that older people are necessarily devalued; after all, there are many calls in bioethics to put an end to the ageism implicit in the allocation of resources, especially in healthcare (Ouchida and Lachs 2015; Nelson 2016; Jecker 2020). In spite of this, when we look at old age and, implicitly, at older individuals through a deficit lens, we tend to deny them decision-making powers; thus, we deny them the capacity of being agents. Our approach shifts the focus away from older adults as simple problematic and passive individuals in both ethical discourse and society at large, towards seeing them as active moral agents with interests, needs, and desires and who can and should be involved in decision-making processes regarding their well-being.

As per usual, there are also exceptions to the general tendency in bioethics to neglect older adults as ethical subjects. For example, Ai-Jen Poo uses the concept of “dignity” as a basis for policies aimed at securing care, justice, and opportunities for the ever increasing proportion of ageing adults, as well as for their caretakers (Poo 2016). Building on the same concept, Jecker argues that there is a midlife bias in bioethics and calls for a focus on old age and respecting the dignity of older individuals, by maintaining their capabilities (Jecker 2020). Still others argue that an appropriate ethical framework for addressing the needs of ageing populations is the ethics of care, for it brings forth the “interpersonal and emotional dimension” of human relationships and individuality (Lloyd 2006). “Recognition” is another concept put forth by Schinkel (2013) who claims that in a just society, what is owed to older adults is precisely recognition of them as individuals, not as a social group. In practical terms, this presupposes authorities’ support for older adults’ autonomy, right to self-determination and social inclusion (Schinkel 2013). All of the above-mentioned frameworks are somewhat distilled in our “respect as paying attention” approach.

Indeed, there have been voices claiming that “dignity means no more than respect for persons or their autonomy,” as such it is unclear whether dignity is substantively different from respect (Macklin 2003). We argue that paying respect means paying attention to the needs and vulnerabilities of particular individuals with the aim of advancing their well-being. Our substantive notion of respect thus stresses the importance of recognizing persons as individuals with the capacity to choose for themselves but who nonetheless have to be acknowledged and understood as embedded in a community which defines and is defined by them. Thus, with this concept of “respect as paying attention” we bring together under the same umbrella the importance of autonomy, the relational dimension of our relationships (ethics of care) as well as the acknowledgement of the importance of paying attention to others (recognizing them). Our approach gives substance to a minimal ethical concept, that of respect for persons, that is widely accepted by everyone as a minimal duty we have towards others and which can, implicitly, gain adherence in policy-making circles. Nonetheless, we do not believe that our approach is a band-aid solution to all of older adults’ problems. Its main weakness lies in the fact that for it to be accomplished, individuals really need to “take respect seriously” by internalizing it as an action-guiding principle. But what does paying respect mean, more precisely?

It is uncontroversial and commonly accepted that we owe one another and even ourselves respect. In fact, respect is such a ubiquitous concept that it often pops up in social and political discourses and is frequently invoked as the basis of all our obligations and duties towards ourselves and the others. Respect mediates our social interactions and structures the social and political arenas, as “it plays an important role in the means by which social justice might be achieved” (Middleton 2004, 228). Many have emphasized that the respect which is due to all persons is a reminder of their intrinsic moral worth (Williams 1973; Murdoch 2013; Rawls 2009; Dworkin 2013; Downie and Telfer 2009). By giving others respect and by receiving respect we develop complex moral relations, which in the end shape our social worlds.

Respect is rich in its different understandings, “fleshed out thickly in the context of different relationships” (Lysaught 2004, 666). The idea that all human beings are to be treated with respect by virtue of their being human beings is not a new idea, being the cornerstone of Kant’s moral philosophy. Kant’s formal structure of morality does not take into account empirical, biological, cognitive, or social differences. It concentrates on what is generic in human beings and was expressed by the Humanity Formulation of the Categorical Imperative: “So act that you use humanity, whether in your own person or in the person of any other, always at the same time as an end, never merely as a means” (Kant 1997, 48). In this sense, the attitude of respect for persons is *morally basic*—all the other moral principles and attitudes towards the others (and towards the humanity in one’s person) are based on it and can be explained in terms of it (Downie and Telfer 2009, 18). But how should this formal understanding of respect guide our behaviours and, implicitly, our systems of justice?

One way of giving substance to the concept of respect is to understand it as a substantial way of paying attention to others or to oneself (in the case of self-respect). For if respect is to be universal, as Kant maintains, then it should not be grounded in one’s preferences or desires. As such, respect emerges from a certain fact that is external to us (Dillon 1992, 108). To apprehend that fact is to pay attention to it, to acknowledge it, to accord it its dues; attention is thus a central element of respect (Murdoch 2013; Dillon 1992; Buss 1999). In fact, Murdoch believes attention “to be the characteristic and proper mark of the active moral agent” (2013, 33). Respect understood as paying attention means, according to Murdoch, not allowing our needs, interests, biases, or even desires to get in the way of appreciating and seeing the others as human beings caught in particular situations, with specific needs and desires (Blum 1986). In the same vein, Bernard Williams argues that respecting persons means that “each man is owed an effort at identification: that he should not be regarded as the surface to which a certain label can be applied, but one should try to see the world (including the label) from his point of view” (1986, 237). Respect thus means paying attention to the subject of respect, considering it in its specificities and particularities and having in view the subjects’ well-being. Paying respect and implicitly attention, the “effort of identification,” as Bernard Williams defined respect, starts from the correct presupposition that we are not individual entities, completely separate from others. But, on the contrary, we become who we are precisely through interaction with others, by being embedded in a network of social relationships which influence and sometimes even define us. As such, respecting another person means “valuing another not just for being a self but for being herself” (Dillon 1992, 121), which means that we should take their well-being into consideration, while “each man is to be (as it were) abstracted from certain conspicuous structures of inequality in which we find him” (Williams 1973, 237). The act of paying attention as a prerequisite of respect does not mean that we should spend our lives trying to understand the specific opinions and beliefs of each and every individual we encounter. Respect presupposes concern towards others, taking their interests into account, helping them when possible, or acting with their well-being in view (Dillon 1992).

Paradoxically, while age-based measures were premised on respect for the lives of older individuals, the lack of government action or interest regarding the impact of these measures on older adults’ well-being demonstrates the contrary. The particular notion of respect fleshed out in this section starts from the presupposition that despite our differences, we all share the basic need to be connected to other people, to be valued, and to feel that we matter. In this sense, paying respect means precisely conveying to the other the fact that their well-being matters, that their interests are acknowledged and taken into consideration. During the COVID-19 pandemic, states imposed measures that have disproportionally affected older adults, as they are more prone to suffer mental and psychological distress due to social isolation and loneliness. In fact, research shows that social isolation increases older adults’ risk of depression and mortality (Barth, Schneider, and Kanel, 2010; Holt-Lunstad, Smith, and Layton, 2010; Santini et al. 2020; Shankar et al. 2017). Moreover, the devaluation of older adults lives in general was manifested in states’ failure to protect and care for older people and their caregivers in care homes and nursing homes as a result of chronic limited investment in quality long-term care across countries. Equally, older adults, living on their own or with their extended families, witnessed a decrease in autonomy during this period. Both categories were marginalized: the former practically exposed to the virus at the margins of the society, out of sight, the latter becoming dependent on the goodwill of other people to provide them with the bare necessities. Which of these persons were asked about or represented in the decision-making processes regarding anti-pandemic policies, even if they were taken into account as categories when giving a rationale to lockdown measures or vaccination priorities? One can point out two forms of injustice which foster ageism (or are fuelled by ageism) during the pandemic: restricting the freedom of movement for those who could take care of themselves and abandoning (mostly by neglect) those in care facilities, the most vulnerable.

## Minimal Ethical Requirement: Respecting Older Persons

The role of the state in protecting the rights of and caring for older persons is not a new issue. Countries have put into place policies that are meant to ensure the financial- and health-related security of older persons (e.g. Social Security and Medicare in the United States). In the last decades, the sustainability of the old age policies of many western countries has been questioned and governments are seeking to find ways to remain sustainable in light of the increasing age of the population. It is becoming more and more clear that care for older adults cannot be delegated solely to families or charity. Because of changes in lifestyles (e.g. women entering and remaining in the workforce) and economic pressure, the time that could be allocated for care, both for children and ageing parents, is becoming more and more scarce. This plight of many adult children sandwiched between caring duties for two generations is discussed in the literature (Boyczuk and Fletcher 2016; Chisholm 1999). Studies highlight how support towards older adults from the welfare state has resulted in more women being able to join and pursue their careers (Haberkern and Szydlik 2010; Haberkern et al. 2015; Wisensale 2005). Thus, moves towards higher expectations of filial duties by the state are both risky and ethically questionable. Families are, theoretically, in the best position to offer attention and care to their elders (de Vries 2020), but we should not assume that this can be taken for granted. This is because there may not be a younger generation of children within a given family in light of childlessness (by choice, infertility, or death of a child) (Albertini and Kohli 2009; Deindl and Brandt 2011). At the same time, previous and current relationships within the family may have been (or are) abusive and neglectful (i.e. past child abuse and recent or ongoing elder abuse), which may nullify opportunities for reallocation of finances, emotional support, and time. Last but not least, younger generations may not be in a position to care for the older parent or may be unwilling to do so either due to individual projects they may have or because caregiving is not agreed to (Jecker 2002). Moreover, many researchers argue that there is no general moral argument which succeeds in showing that children have a duty to care for their parents (Daniels 1982; Stuifbergen and Van Delden 2011). Due to space constraints, we will not delve into this debate. Nonetheless, it is important to stress that even if one could possibly build a convincing argument to show that children do have certain obligations to care for and help their older parents, this could in no way guide public policy aiming to secure justice for older generations, because it would leave aside the childless among older persons.

As such, respect for older adults, understood as paying attention to their interests with the end aim of furthering their well-being, cannot be delegated solely to their families. This means that it is up to states to secure and ensure respect for older adults, if indeed respect is to be seen as a morally basic attitude towards the others that each and every human being is due. In fact, states have duties to treat *every* human being with respect, regardless their age, social status, sex, or any other contingent attribute. But it is precisely older individuals’ well-being which was affected during the pandemic the most, either as a result of the adoption of risk-mitigation measures or the entrenchment of ageist attitudes could result in the complete alienation of older adults.

The overall effect of this pandemic is the highly increasing level of alienation, understood as a disengagement of individuals from social processes and phenomena that shape their social worlds (Connolly 2020; O’Brien 2020; Rowe, Ngwenyama, and Richet 2020). Of course, alienation is not felt only by older individuals but in their case, it manifests itself even more deeply. As a subjective feeling, the estrangement perceived by older adults cannot and could not be fully addressed by formal institutions of care. Loneliness can be diagnosed by a physician, but the cure is not in the hands of medicine, drugs, etc., which can be palliative but not effective at treating the cause. The trajectory of loneliness is degenerative: the older you get, the lonelier you might be.

## In Place of a Conclusion: A Sketch of Possible Redress Measures

The alienation and injustices experienced by older adults need redressing to assure respect. In most Western liberal societies governmental help schemes were put in place for certain categories (such as business owners, employees in certain sectors, parents), as a means of redress for the consequences of the lockdown and of the slowing down of economies. But that was not also the case of older adults. Redress should be seen as corrective not retributive (Vică 2015); it has to correct a state of affairs where rights were trespassed or their exercise was restrained (even if this was justified on the grounds of population or public ethics principles). Here we sketch some examples of redress measures states and civil society have to put into practice in order to restore due respect, reduce alienation, and promote a productive, positive image of ageing. An exceptional measure of redress was indeed adopted by establishing priority in vaccination for older adults. But this measure is not enough; more should be done in order to reintegrate older adults into social and political life and to offer them the means to be autonomous and to develop positive social relations. This could be accomplished without too many costs by, for example, reforming nursing homes, promoting informal networks of support, countering the ageist rhetoric in public messages and mass-media, and bridging the digital divide.

Residents of nursing homes account for the biggest proportion of deaths amongst different social categories during the COVID-19 pandemic (Ouslander and Grabowski 2020). This situation was determined not only by the “vulnerability” of older adults in the face of the virus but also by the lack of preparation, staffing shortages, and lack of resources (Fallon et al. 2020). Moreover, due to the institutionally separate nature of nursing homes and to physical distancing measures, residents have been exposed to abuse and violence (D’cruz and Banerjee 2020). Some nursing homes were even accused of covering up the number of deaths (Ouslander and Grabowski 2020). But this is not necessarily new, as the problem of the low quality of care in nursing homes long precedes the pandemic. In general, medical professionals and governmental authorities are not quite interested in “research, recruitment incentivization and quality improvement in nursing homes” (Fallon et al. 2020, 391). This shows just how deep disregard for the lives of older adults runs in most Western societies. If this pandemic made something clear, it is just how much we need to reform the “nursing industry.” Indeed, our framework is useful at this point not for showing how things should be changed but for stressing how these institutions failed and still fail to treat residents with the respect they are due.

States should also promote informal networks of support, such as some of those that arose during the pandemic in order to provide older adults with whatever they needed (Vervaecke and Meisner 2021). The COVID-19 pandemic created the conditions for a natural social experiment: these networks were grassroots and arose as a civic and moral response to the lockdown and states’ lack of support and action to provide older adults with food, medicine, or other necessities (Fraser et al. 2020). These networks were horizontal, composed of family members, friends, and other informal groups, like neighbours, NGO volunteers, physicians and caregivers, and even people who coordinated online to create awareness and to support older adults. As peer networks, they have no central command or authority but, by their nature, are dynamic and flexible enough to orientate around different nodes, i.e. people in need of care and attention. The volatility of these networks is real, but their fluidity makes them more punctually efficient than formal institutions are. One model of informal support network is to be found in the case of drug and alcohol recovery programmes. In this case, the network works not only due to the generic equality it imposes but also because it is not compulsory or binding. To be under the protection of formal institutions presupposes an official assessment, bureaucratic and impersonal; instead, informal support is offered voluntarily, it is personal and addresses the specific issues of each person.

There is always a risk for informal support networks to degenerate into “care mongering”—patronizing sentiments towards older adults stemming from compassionate ageism (Vervaecke and Meisner 2021). In order to mitigate this risk, states as well as civil societies, should counter any ageist rhetoric by promoting more accurate messages about older adults and ageing. So, a further redress measure is the reshaping of public discourses so as to recognize the diversity and opportunities that old age presents. For instance, governmental agencies should avoid speaking about older adults, especially in public health messages, as if they were a homogenous category; in the same vein, they should resist the impulse to conflate old age with frailty and vulnerability.

Another redress measure that states have to put in place consists in bridging the digital divide between older adults and younger generations, by promoting digital, virtual, and even artificial support systems (DiMaggio and Harggitai 2010). The barriers that older adults face in using new technologies, such as lack of exposure to them or the high costs and limited support in learning how to use them (among many others), prevent them from accessing new technologies that could substantially improve their lives, by enabling knowledge gain, closer family relations, and overall connection to society (Delello and McWhorter 2017; Friemel 2016). Some technologies are already deployed in care homes and other health facilities (Bemelmans et al. 2012). Their role is not to replace human care but to enhance the quality of care. In cases of social loneliness or relational isolation, which is a form of alienation, the lack of quality friendships or family connections can be countered by a mix of involvement in informal networks and the presence of artificial companions, which can take a large range of forms and embodiments (not limited to androids). But we should keep in mind that not all psychological needs can be satisfied with one social relationship. Attachment, sense of worth, or happiness are produced within a diversity of strong and weak ties. Moreover, social interactive care robots should be deployed with care to the various ethical problems they raise (Sharkey and Sharkey 2012).

In a way, because of the deeply entrenched ageism in our societies, we feel that we have done enough for older adults already, by going into general lockdown or by “sacrificing our economies” in exchange for their lives. We feel as if we fulfilled our duties towards them, and any more action would be supererogatory. But the feeling of powerlessness and sometimes even that of a loss of authenticity (due to the regulations which imposed a strict, specific behaviour), cannot be compensated for by the probabilistic guarantee of being physically protected against the virus. We all are bound by the obligations to pay respect which does not mean simply to refrain from doing harm—it is not only a negative duty. Indeed, abstaining from harming is a necessary, but not sufficient, condition to instantiate respect. Respect also encompasses the obligation to ensure, protect, and foster all the necessary conditions for human beings to pursue their own ends and good, to be autonomous and able to develop fulfilling social relations.
